# Supplementary material for: Differential Expression of Lonp1 Isoforms in Cancer Cells
Source: Cells. 2022 Dec 6;11(23):3940. doi: 10.3390/cells11233940 (PMC9739308; doi:10.3390/cells11233940)
Supplement: Supplementary file 1 [file cells-11-03940-s001.zip › cells-1924032-supplementary.pdf]

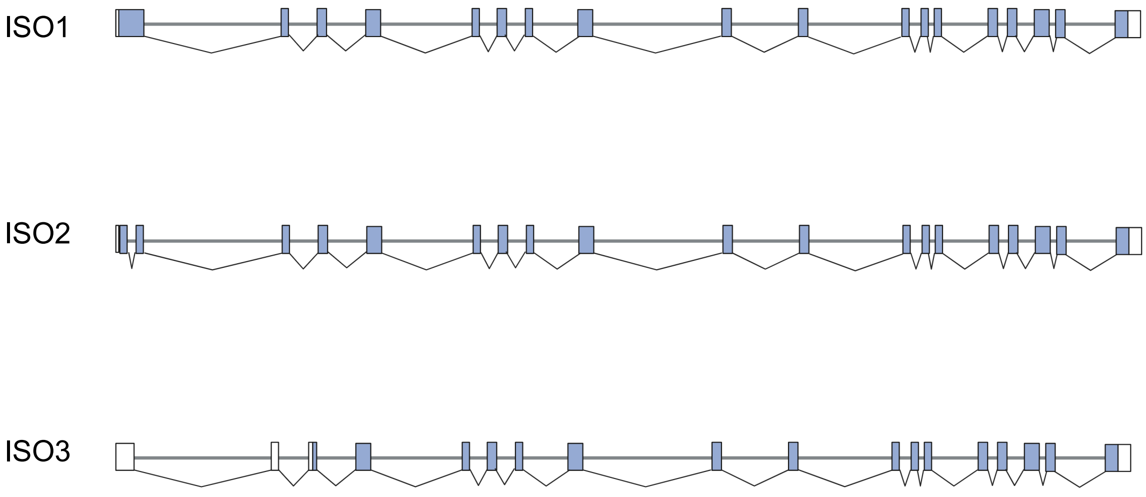

Supplementary Figure S1. Exon/intron organization of Lonp1 gene. The exons included in the ISO1, ISO2 and ISO3 mRNAs are shown in light blue, while the exons excluded are shown in white.

Suppl. Fig. S1

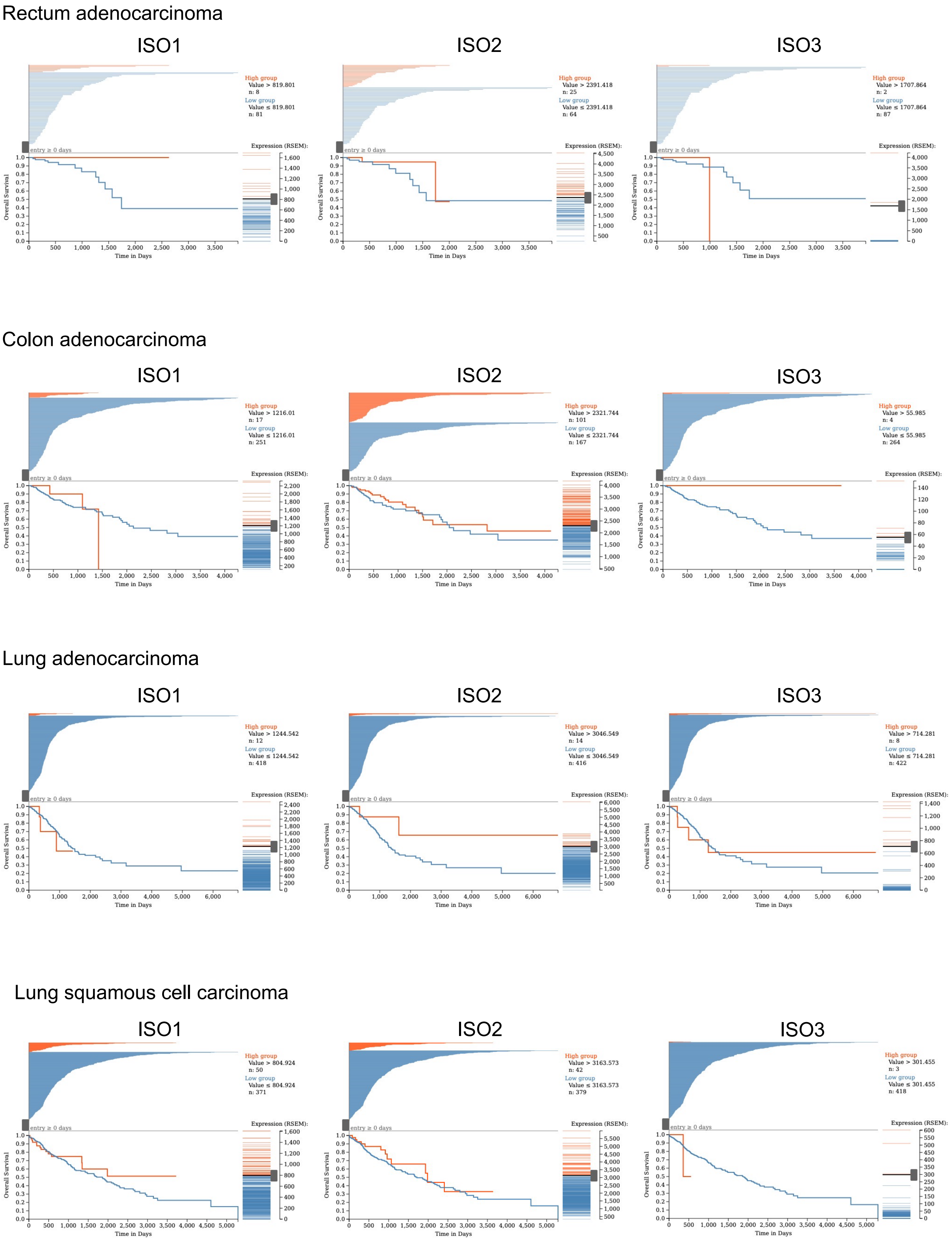
Rectum adenocarcinoma
ISO1
High group
Value > 819.801
n: 8
Low group
Value ≤ 819.801
n: 81
ISO2
High group
Value > 2391.418
n: 25
Low group
Value ≤ 2391.418
n: 64
ISO3
High group
Value > 1707.864
n: 2
Low group
Value ≤ 1707.864
n: 87
Colon adenocarcinoma
ISO1
High group
Value > 1216.01
n: 17
Low group
Value ≤ 1216.01
n: 251
ISO2
High group
Value > 2321.744
n: 101
Low group
Value ≤ 2321.744
n: 167
ISO3
High group
Value > 55.985
n: 4
Low group
Value ≤ 55.985
n: 264
Lung adenocarcinoma
ISO1
High group
Value > 1244.542
n: 12
Low group
Value ≤ 1244.542
n: 418
ISO2
High group
Value > 3046.549
n: 14
Low group
Value ≤ 3046.549
n: 416
ISO3
High group
Value > 714.281
n: 8
Low group
Value ≤ 714.281
n: 422
Lung squamous cell carcinoma
ISO1
High group
Value > 804.924
n: 50
Low group
Value ≤ 804.924
n: 371
ISO2
High group
Value > 3163.573
n: 42
Low group
Value ≤ 3163.573
n: 379
ISO3
High group
Value > 301.455
n: 3
Low group
Value ≤ 301.455
n: 418
entry ≥ 0 days
Expression (RSEM):
Overall Survival
Time in Days

Suppl. Fig. S2

Cervical squamous cell carcinoma and endocervical adenocarcinoma

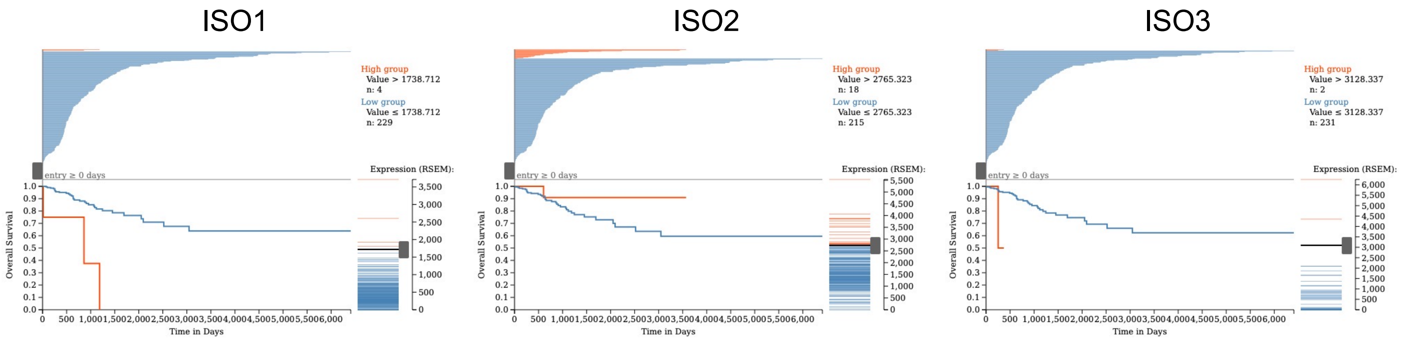

Bladder urothelial carcinoma

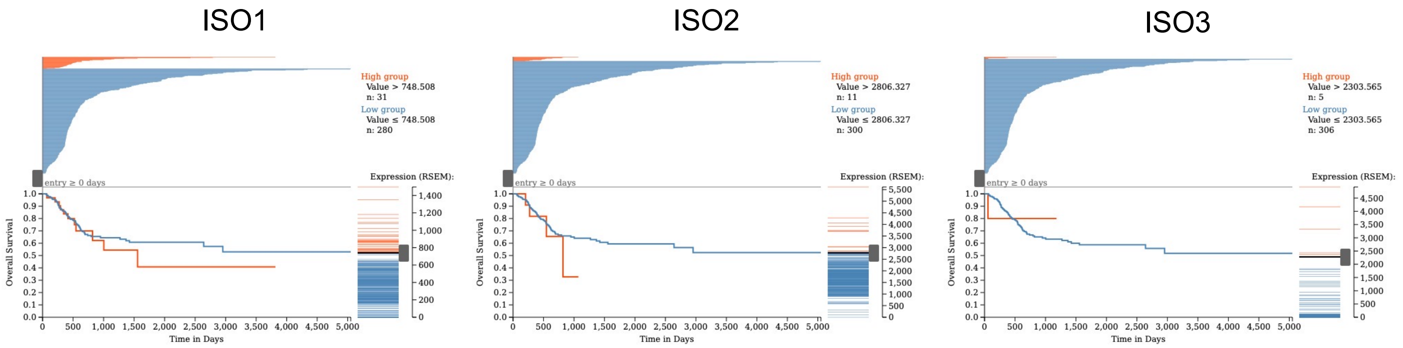

Prostate adenocarcinoma

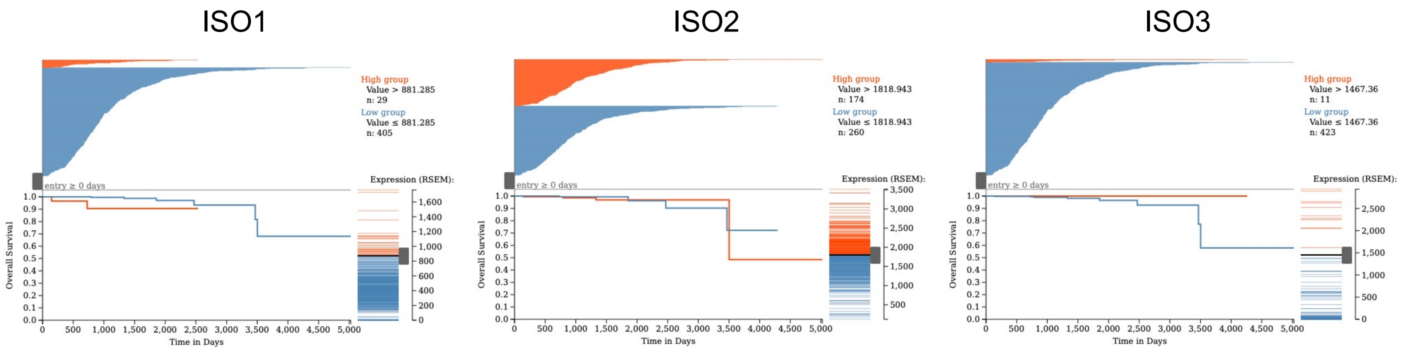

Breast invasive carcinoma

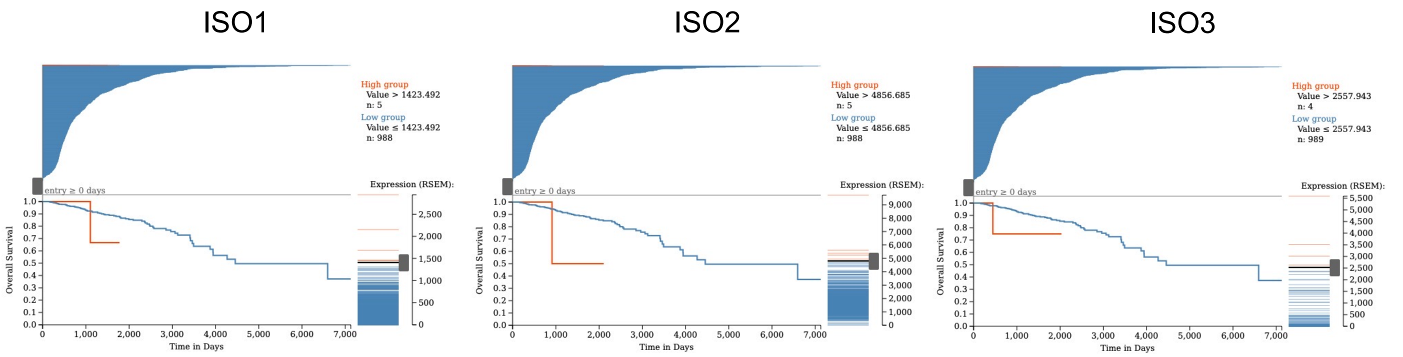

Suppl. Fig. S2

Head and neck squamous cell carcinoma

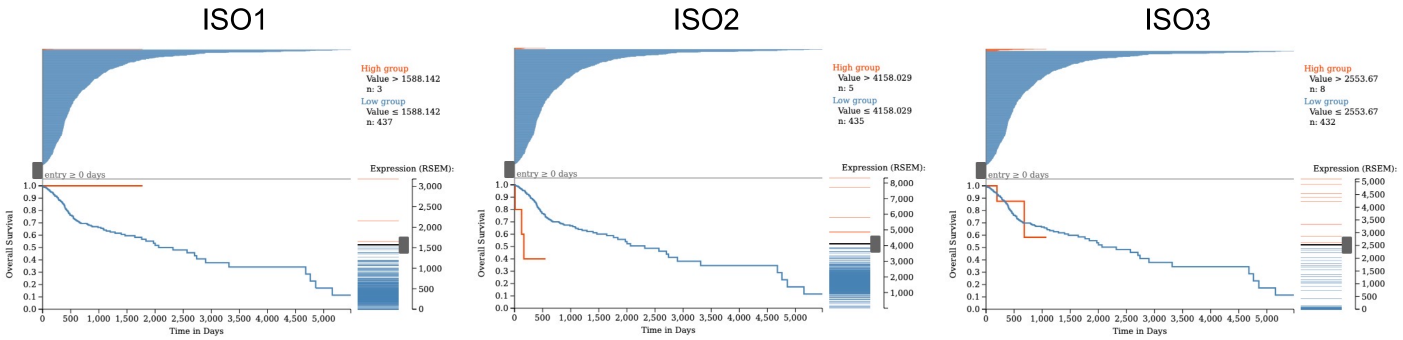

Kidney renal clear cell carcinoma

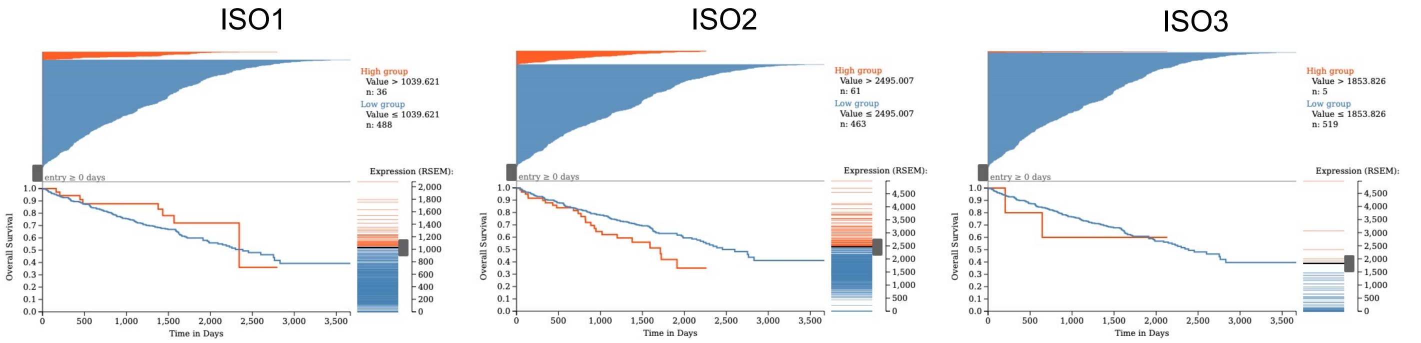

Kidney renal papillary cell carcinoma

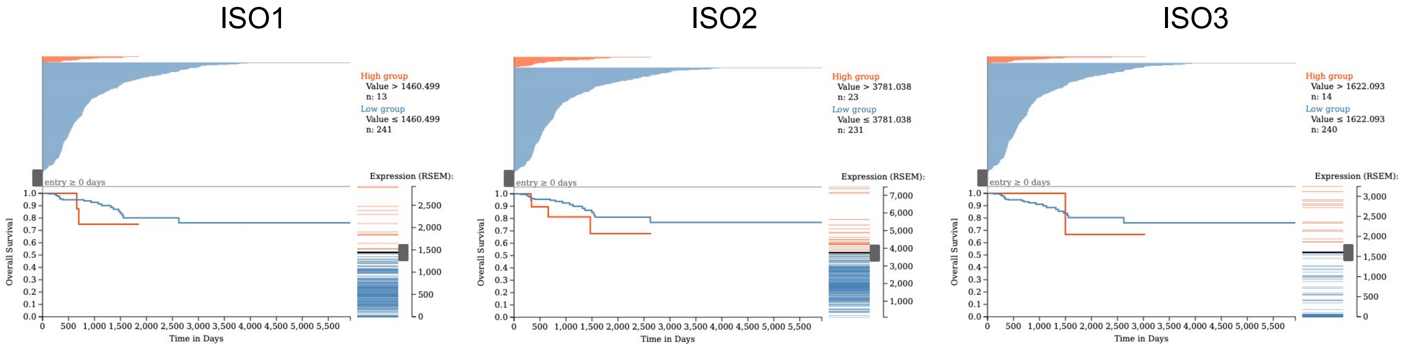

Supplementary Figure S2. Kaplan-Meier overall survival curves of patients stratified on the expression of Lonp1 isoform-1, isoform-2, and isoform-3 in different primary solid tumours.

Suppl. Fig. S2

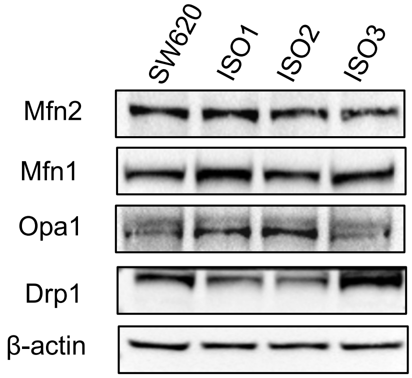

Supplementary Figure S3. Effects of Lonp1 isoform-1, isoform-2, and isoform-3 overexpression on mitochondrial dynamics.

Representative immunoblots showing Mfn2, Mfn1, Opa1, and Drp1 expression in SW620 and cells overexpressing Lonp1 isoform-1, isoform-2, and isoform-3.

Suppl. Fig. S3
